# Perceived benefits of geriatric specialty telemedicine among rural patients and caregivers

**DOI:** 10.1111/1475-6773.14055

**Published:** 2022-09-14

**Authors:** Eileen M. Dryden, Meaghan A. Kennedy, Jennifer Conti, Jacqueline H. Boudreau, Chitra P. Anwar, Kathryn Nearing, Camilla B. Pimentel, William W. Hung, Lauren R. Moo

**Affiliations:** ^1^ Center for Healthcare Organization and Implementation Research Department of Veterans Affairs (VA) Bedford Healthcare System Bedford Massachusetts USA; ^2^ New England Geriatric Research, Education, and Clinical Center VA Bedford Healthcare System Bedford Massachusetts USA; ^3^ Department of Family Medicine Boston University School of Medicine Boston Massachusetts USA; ^4^ Eastern Colorado VA Geriatric Research Education and Clinical Center Aurora Colorado USA; ^5^ Division of Geriatric Medicine University of Colorado Anschutz Medical Campus Aurora Colorado USA; ^6^ Department of Public Health, Zuckerberg College of Health Sciences University of Massachusetts Lowell Lowell Massachusetts USA; ^7^ Bronx Geriatric Research Education and Clinical Center James J. Peters VA Medical Center Bronx New York USA; ^8^ Department of Geriatrics and Palliative Medicine Icahn School of Medicine New York City New York USA; ^9^ Department of Neurology Harvard Medical School Boston Massachusetts USA

**Keywords:** geriatrics, referral and consultation, rural, telemedicine, veterans health

## Abstract

**Objective:**

Explore the perceived benefits of a Veterans Health Administration (VHA) geriatric specialty telemedicine service (GRECC Connect) among rural, older patients and caregivers to contribute to an assessment of its quality and value.

**Data Sources:**

In Spring 2021, we interviewed a geographically diverse sample of rural, older patients and their caregivers who participated in GRECC Connect telemedicine visits.

**Study Design:**

A cross‐sectional qualitative study focused on patient and caregiver experiences with telemedicine, including perceived benefits and challenges.

**Data Collection:**

We conducted 30 semi‐structured qualitative interviews with rural, older (≥65) patients enrolled in the VHA and their caregivers via videoconference or phone. Interviews were recorded, transcribed, and analyzed using a rapid qualitative analysis approach.

**Principal Findings:**

Participants described geriatric specialty telemedicine visits focused on cognitive assessments, tailored physical therapy, medication management, education on disease progression, support for managing multiple comorbidities, and suggestions to improve physical functioning. Participants reported that, in addition to prescribing medications and ordering tests, clinicians expedited referrals, coordinated care, and listened to and validated both patient and caregiver concerns. Perceived benefits included improved patient health; increased patient and caregiver understanding and confidence around symptom management; and greater feelings of empowerment, hopefulness, and support. Challenges included difficulty accessing some recommended programs and services, uncertainty related to instructions or follow‐up, and not receiving as much information or treatment as desired. The content of visits was well aligned with the domains of the Age‐Friendly Health Systems and Geriatric 5Ms frameworks (Medication, Mentation, Mobility, what Matters most, and Multi‐complexity).

**Conclusions:**

Alignment of patient and caregiver experiences with widely‐used models of comprehensive geriatric care indicates that high‐quality geriatric care can be provided through virtual modalities. Additional work is needed to develop strategies to address challenges and optimize and expand access to geriatric specialty telemedicine.

**What is known on this topic**

- Telemedicine has the potential to increase access to geriatric specialty care, particularly for older adults living in rural areas.
- While there was a rapid expansion of telemedicine during the COVID‐19 pandemic, there is limited research on the quality of geriatric care delivered via telemedicine.
- Age‐Friendly Health Systems and the Geriatric 5Ms are models of comprehensive, high‐quality geriatric care, but it remains unclear how these models translate to virtual care delivery.

**What this study adds**

- Perceived benefits of GRECC Connect (geriatric telemedicine) included positive impacts on patients' health and coordination of care in addition to providing support for and building the confidence of patients and caregivers.
- Patients' and caregivers' perceptions of the content and benefits of the GRECC Connect geriatric telemedicine service are well‐aligned with the Age‐Friendly Health Systems model and Geriatric 5Ms framework.

## 1 INTRODUCTION

While many people in the United States face challenges accessing health care services, it is especially true for the fifth of the population who live in rural areas.^1^, ^2^, ^3^ Compared to urban populations, those in rural communities tend to be older and in poorer health^4^ and face numerous barriers to health care access, including a paucity of hospitals, clinics, and health care clinicians and a lack of transportation options.^1^, ^2^, ^5^, ^6^

Telemedicine is a promising solution to many of these barriers.^7^ Rural, older adults, in particular, could benefit from telemedicine given the shortage of geriatric specialists in rural areas^6^, ^8^ and likelihood of this population facing barriers to travel related to mobility, vision, and cognitive challenges.^9^ Although there has been recent rapid growth in telemedicine catalyzed by COVID‐19 restrictions on in‐person care,^10^ telemedicine utilization has historically been low,^11^, ^12^ particularly among older adults.^13^ For some, the ritual of in‐person care^14^ is closely tied to high‐quality care and is believed to provide the basis for building patient‐provider rapport and medical trust.^15^ This leads some patients and physicians to question if high‐quality care can be provided virtually.^13^, ^16^, ^17^, ^18^

Many of the same characteristics that make travel to in‐person medical visits difficult for older adults (e.g., hearing, vision, and cognitive impairment) can also make it more challenging to access care virtually.^19^ Furthermore, older adults may be less likely to have the technological devices or literacy needed to successfully participate in virtual medical visits.^20^, ^21^, ^22^ This has been the impetus for research focused on acceptability,^13^, ^23^ usability,^19^ and facilitators and barriers to implementation^24^ of geriatric telemedicine. While these studies of feasibility are necessary, the Technology Acceptance Model suggests that perceived usefulness among end‐users is also critical for determining the value of an alternative model of care delivery.^25^, ^26^ Two systemic reviews support the general feasibility and acceptability of telemedicine among older adults, but call for more research to be done on the quality of telemedicine care for this population,^27^ especially for those living in rural areas since the existing, limited data are mixed.^28^ A recent study focused on perceived benefits of geriatric telemedicine by patients and clinicians during the COVID‐19 pandemic reported divergent views on its usefulness and concluded that quality of care for some older adults suffered.^16^ It is challenging to tease out participants' perceptions of the content of the visit versus the modality—indeed, they sometimes overlap—yet this is the type of understanding that is needed to create meaningful post‐pandemic policies that will sustain or even expand high‐quality telemedicine for older adults in the future.^10^, ^29^

Judgments of quality of care may depend on the type of health care visit. Age‐Friendly Health Systems aim to provide older adults with evidence‐based care aligned with their health care preferences and goals, making this model appropriate for assessing geriatric care visits.^30^ Many experts consider the 4Ms framework of the Age‐Friendly Health Systems model— Medication, Mentation, Mobility, and what Matters most—as well as Multi‐complexity as a 5th M in some expanded models^31^ —to be an essential part of comprehensive, quality geriatric health care.^32^ Using a 5Ms framework means providing care for older adults that aligns with their goals and preferences and focuses on medications (e.g., polypharmacy concerns, how their use interacts with the other 4Ms), mentation (e.g., mood and cognition), mobility (e.g., gait and ability to safely move about their environment), and multi‐complexity (i.e., acknowledging and addressing the often complex intersectionality of multiple chronic and acute ailments and social context).^33^ To our knowledge, there are no published studies on how this framework translates to the virtual environment, though some literature outlines its potential.^34^

The Veterans Health Administration (VA) has long relied on telemedicine to care for 2.7 million rural Veterans,^35^ among whom more than 55% are aged 65 or older.^36^ The GRECC Connect program, started in 2014 and funded by the VA Office of Rural Health, connects geriatric interdisciplinary teams from 15 hub sites in urban areas with older veterans in rural areas through secure VA‐approved videoconferencing platforms. Because GRECC Connect delivers geriatric care visits based on the needs of primary care teams in rural areas, the majority of the visits are related to the assessment or management of cognitive impairment or dementia; however, comprehensive geriatric assessments, management of complex comorbidities, and other specialized geriatric care are delivered remotely as well. (See Pimentel et al. 2019 for a full description of the GRECC Connect program.) To explore the extent to which high‐quality geriatric care can be provided to rural, older adults through telemedicine, we interviewed older, rural veterans and their caregivers about the content and quality of their GRECC Connect telemedicine visits.

## 2 METHODS

### 2.1 Study design

We conducted a cross‐sectional qualitative evaluation with patients and caregivers who had participated in at least one GRECC Connect telemedicine visit within a 3‐month period prior to the interview.

### 2.2 Study team

The overall evaluation team consisted of nine VA researchers with expertise in program evaluation, qualitative methods, implementation science, and geriatric medicine, as well as a Veteran research consultant with experience providing technical assistance to older adults. The subgroup of this team that conducted the interviews was headed by an anthropologist (Eileen M. Dryden) with over 25 years of experience leading qualitative research and evaluation studies and included three master's prepared health services researchers with substantial experience in public health and qualitative methods (Chitra P. Anwar, Jacqueline H. Boudreau, and Jennifer Conti). For the analysis phase, the group was expanded to include a fifth team member, a physician‐scientist (Meaghan A. Kennedy), who provides primary care for and conducts mixed‐methods research focused on older adults.

### 2.3 Setting

In December 2020, we sent letters to the clinical leads of seven of the 15 GRECC Connect hubs to serve as recruitment sites. We selected hubs that were geographically diverse and had a high enough volume of patient encounters to support recruitment.

### 2.4 Interview participants

Interviewees were recruited from lists of patients who completed GRECC Connect telemedicine visits between December 2020 and March 2021. We defined “telemedicine visits” as (1) video visits conducted between a remote geriatrics specialist and a patient at home (VA Video Connect [VVC]) or at a community‐based VA clinic (Clinical Video Telehealth [CVT]); or (2) telephone visits conducted between a remote geriatrics specialist and a patient at home. Prior to initial contact, three team members briefly reviewed each patient's electronic health record (EHR) to determine the telemedicine visit date and modality and screen for eligibility. Patients were eligible for participation if they were ≥65 years old, resided in a rural area (rural–urban commuting area code [RUCA] >1),^37^ participated in a video or telephone GRECC Connect appointment between December 2020 and March 2021 and spoke English as their primary language. We aimed to interview 30 participants; a sample large enough to reach “meaning saturation,”^38^ that is, to provide a range of experiences and sufficient rich, detailed qualitative data to understand the topic of study, yet small enough to feasibly implement within the study scope and timeframe. Female and non‐White patients were contacted first in an effort to oversample these participants in a largely White, male population of older, rural Veterans. Gender and race were identified through patients' EHR.

### 2.5 Recruitment

We recruited our sample via mailers and then by telephone. Prior to interviews, an evaluation team member performed a brief phone screening with the patient and/or the patient's caregiver to confirm recall of a specific GRECC Connect telemedicine appointment (“index appointment”) around which to ground the interview. To enhance their ability to recall the appointment, we chose to limit recruitment to individuals with an index appointment within the 3‐month period prior to the interview. Once participants consented to participation, a study team member performed a detailed chart abstraction of the patient's EHR using a structured template. Information from chart abstractions was integrated into a visual appointment summary that depicted, in lay language, the content discussed and outcomes (e.g., referrals or medication changes) of the index appointment.

### 2.6 Data collection

Four experienced qualitative researchers (Chitra P. Anwar, Jacqueline H. Boudreau, Jennifer Conti, Eileen M. Dryden) conducted semi‐structured qualitative interviews with participants via videoconference or phone. In cases where patient participants had some degree of cognitive impairment (e.g., dementia), we interviewed the patient‐caregiver dyad (“the dyad”). While the interview included questions concerning experience with the technological aspects of the visit (manuscript in progress), the questions that are the focus of this manuscript explored patient and caregiver perceptions of visit content (i.e., the focus of and activities that occurred during the visit) as well as perceived benefits, challenges, and impact. This included questions about changes the dyad experienced as a result of the visit (e.g., in diagnosis, medications, activities, support services), including differences in health, physical or emotional well‐being, and ability to take care of things at home. We also asked about satisfaction with the visit and their recommendations to improve the quality of the visit. A shareable visual appointment summary was used to facilitate recall and provide a shared conversational reference for the interviewer and interviewee(s).^39^

### 2.7 Data analysis

Interviews were recorded with permission and transcribed verbatim; transcripts were then reviewed to verify accuracy. All interviews were analyzed by a team of five experienced qualitative researchers (Chitra P. Anwar, Jacqueline H. Boudreau, Jennifer Conti, Eileen M. Dryden, Meaghan A. Kennedy) using a rapid qualitative analysis approach.^40^, ^41^ Once all interviews were completed, analysts summarized individual interviews using a structured template, meeting regularly to develop consensus about domains, which included a priori and emergent domains from the semi‐structured interview guide and interview content. To develop consensus and consistency in the application of the structured template, two interviewers summarized two initial transcripts and three reviewers reviewed the summaries. Ten additional interviews were summarized in rotating pairs or triads. The remaining transcripts were summarized individually, with all analysts meeting regularly to resolve uncertainties or refine content domains. Summary templates were condensed into a single matrix where each row represented a participant/dyad and each column a domain, to allow for summarizing findings within and across domains. The analysis team identified common, unique, and salient themes in the data and reviewed them, along with illustrative quotes, with the larger evaluation team. The larger team included GRECC Connect leadership and physicians, as well as a primary care provider, and other evaluators of geriatric services. This group of content and methodological experts provided context for and helped to interpret the findings.

### 2.8 Ethics

This work was determined to be quality improvement/evaluation by the VA Bedford Healthcare System Institutional Review Board (IRB), and therefore not subject to IRB approval and oversight as human subjects research.

## 3 RESULTS

Of the seven hub sites invited to participate in the evaluation, one site declined, citing a lack of staffing capacity to generate patient lists from which the evaluation team could recruit. We sent mailers to 110 individuals associated with the remaining six hub sites; 19 people called to opt out of recruitment. We attempted to contact the 91 remaining patients by telephone but were unable to reach 31 of them. Of those we reached, 25 declined to participate and we were unable to find a time to schedule an interview with five of them. Our final sample included 30 Veterans. Interviews ranged from 45 to 60 min in length. Fifty‐seven percent were conducted by VVC, a VA‐approved videoconferencing platform, 40% were conducted by telephone, and one was conducted by a combination of VVC and telephone.

### 3.1 Participants

Patient participants (*n* = 30) were all male; 96% were White, non‐Hispanic, one participant was White, Hispanic, and one was unknown. Mean participant age was 75 years. Most participants' index GRECC Connect visits were initiated to address cognitive impairment. At least two‐thirds participated in visits using a video modality. Most index visits (23/30) included a caregiver and were not the patient's first telemedicine encounter. (See Table 1 for Index visit characteristics.) Nineteen (63%) of the interviews were conducted with dyads.

**TABLE 1:** Telemedicine visit characteristics

GRECC connect visit characteristics	Participants (*n* = 30)	
	Frequency	Percentage
Reason for visit		
Initial cognitive impairment	16	53%
Follow‐up cognitive impairment	10	33%
Other^a^	4	13%
Visit modality		
Phone	7	23%
VA video connect (VVC – video to home)	8	27%
Clinical video telehealth (CVT – video to rural community clinic)	12	40%
CVT per medical record; described by patient as phone visit	2	7%
Combination of phone and VVC	1	3%
First experience with telemedicine		
Yes	2	7%
No	27	90%
Unknown/no data	1	3%
Present in visit		
Patient	7	23%
Patient and caregiver	22	73%
Caregiver only	1	3%

^a^ Falls, physical therapy, medication consultation after stroke, evaluation of sleep hygiene.

#### 3.1.1 Visit satisfaction

Patients and caregivers were largely satisfied with GRECC Connect, and many said they would recommend GRECC Connect to other patients. Overall, participants found the GRECC Connect clinicians (both individually and in teams) to be thorough, professional, competent, and responsive to their needs.

*“I think the care is great. I mean, we're very pleased with it. I feel that, you know, that's where the focus is on the geriatric side of giving good care. You know, just consistent care where it's comfortable‐ and a good quality of life and that's really what we're wanting is the quality of life.”* Caregiver J‐Site 3.

Some participants voiced dissatisfaction owing to unmet expectations related to the purpose or the process of the visit. For example, a dyad was skeptical that an Alzheimer's diagnosis could be determined based on “a few questions” conducted virtually and worried whether the visit purpose was inappropriate for telemedicine. Ultimately, the process of doing this assessment and making the diagnosis was unsatisfactory for this dyad.

*“I thought maybe they would do more testing, asking questions [like], you know, “remember this and remember that,” and that did not happen. They just listened to what we had to say [and] then diagnosed him with this. (…) I just need to know how they came to this conclusion without any testing. This is the hard part for me to understand*.” Caregiver E – Site 1.

#### 3.1.2 Benefits associated with the visit

Patients and caregivers described a number of perceived benefits of GRECC Connect telemedicine: improved health for the patient; increased knowledge and confidence; and feeling empowered, hopeful, and supported.

### 3.2 Improved health for the patient

Patients and caregivers reported improvements in physical and cognitive health due to medication changes (i.e., prescribing, deprescribing, changing dosage); education about the importance of certain treatments and their impact on memory (e.g., leading to more consistent use of a CPAP machine and oxygen); more consistent access to tailored physical therapy; and safer environments due to reduced fall risks in the home. Improved health outcomes attributed directly to these activities included lower blood pressure, better quality sleep, improved memory, fewer falls, less confusion, and less anxiety. The following was noted by a caregiver and corroborated by the patient:

*“I really think since he's been taking this pill he's resting at night better. His anxiety is less and he seems to be remembering things a little better (…) I think that him resting‐ not just, you know, sleeping but really resting – I think that's helped him.”* Caregiver B‐Site 2.

Some caregivers noted changes in the patient's health translated to better health for them as well. For example, some noted that once the patient was sleeping better, they slept better, too.

### 3.3 Increase in patient and caregiver knowledge and confidence

Participants noted the evaluations, diagnoses, updates on the progression of illness, and education around managing symptoms increased their knowledge and confidence. For some, these visits confirmed what they felt they already knew but they appreciated the information and felt validated. Others said they really had ‘no idea what was going on’ so a diagnosis during the telemedicine visit provided a new understanding. Many described getting clarity around the patient's mental and physical state during the visit, and for some, that put them at ease.

*“They solved a lot of my problems dealing with him. There are so many things they helped me with (….) (For example,) the doctor gave me a good way to deal with him when he gets upset. Told me to touch him lovingly, tell him, “Hey, I know you're upset. It'll be okay,” and it works.”* Caregiver C‐Site 1.

### 3.4 Increase in patients' and caregivers' feelings of empowerment and hope

Both patients and caregivers described feeling empowered and hopeful due to increased knowledge about their condition and having more tools/resources (e.g., medications, recommendations for activities and programs, and options for enrolling in clinical trials). Many shared that they liked having suggestions of something to do to help slow the progression of the disease.

*“Both [in‐person and CVT visits] are very informative and very helpful (…) so you make your own decisions*… *If you need to do something, then you can. It empowers you to do something.”* Patient B‐Site 3.

Even those who were unable to participate in the suggested activities or had yet to experience any changes in their health indicated that receiving actionable knowledge instilled hope.

*“I think (my memory) is still pretty much the same. The fact that I wasn't able to do the exercise thing on the tablet or the computer, kind of everything stayed on the same plateau. (However,) it gets me hopeful. I kind of look forward to improving (…) Before, I was just like ‘oh, it is what it is’ type of an attitude and now there's hope.”* Patient A‐Site 1.

### 3.5 Patients and caregivers feel supported

Both caregivers and patients described feeling very supported after participating in GRECC Connect visits. GRECC Connect clinicians shared contact information of people to call with questions or requests for support services; ordered needed items like incontinence undergarments and mattress covers, which were greatly appreciated; and advocated for the patients by facilitating expedited referrals and coordinating care. One caregiver expressed that this made them feel like they were part of a team. Knowing there was someone to call if they needed something also made many of them feel more confident.

*“I don't feel so alone now, and I think that he's doing a little better*.” Caregiver E‐Site 4.

Many patients and caregivers described GRECC Connect clinicians spending time simply listening to their experiences, fears, and concerns about their mental and physical health, answering questions, and sharing their knowledge and expertise. These actions made patients and caregivers feel validated, confirmed their suspicions, made them feel supported and cared for, helped normalize their experience, and assured them that “they were doing things right.”

*“It really makes us both feel better (…) It's just helpful to know that somebody's out there that cares, I guess.”* Caregiver B‐ Site 1.

#### 3.5.1 Alignment with the geriatric 5Ms

During the interviews, participants were asked about the focus of activities that occurred during the telemedicine visit. While these are discussed above along with associated perceived benefits, it is notable that the most salient aspects of the visits aligned with the Age‐Friendly Health Systems 4Ms framework (plus the 5th Multicomplexity domain). See Table 2 for telemedicine visit content organized by the Geriatric 5 M domain.

**TABLE 2:** Geriatric telemedicine visit content aligned with 5Ms domain

5 M domain	Aligned visit focus from interviews	Illustrative quotes
Medication	*Deprescribing* *Prescribing* *Medication dosage adjustment* *Medication review* *Education around medication usage*	*“(The GRECC Connect clinician) helped me understand the options for medication (…) If his memory continues to deteriorate, then I need to contact them to get a prescription for one of the medications. She also helped us understand to be careful with certain medications (…)* Caregiver D‐Site 2
Mentation	*Agitation* *Anxiety* *Depression* *Memory loss* *Cognitive and mental health evaluation* *Recommendations around activities to improve mood and cognition*	*“Well, on the Zoloft he's not pacing as much (…) He still has periods where he gets, like, you know, anxious and stuff, but it's not as bad”* Caregiver E‐Site 5 *“I think it's given me a little more confidence, well I know now I've got connection with the Memory Center so if I need to reach out for something I know I can. (…) We, I and Dad and my siblings, collectively we have a better understanding of the situation and now have contacts if I need assistance. I can call (the GRECC Connect provider) or I can call the (primary care provider) and get him on some medications that might help, (…) – we had none of this (before) because his memory had never been evaluated to any significant extent previously. So this is helpful.”* Caregiver D‐Site 2
Mobility	*Fall prevention* *Physical therapy* *Home safety evaluation* *Durable medical equipment* *Education around exercises and activities to maintain and improve mobility*	*“They've made recommendations for grab bars in the shower and the ramp for outside. (…) at one point they looked at what type of walker he had and they upgraded his walker for his condition. So they watch him and how he executes his exercises and make suggestions of how he can do it more affectively or more safely. The big thing is safety for him because he falls frequently.”* Caregiver A‐Site 6
What matters most	*Planning care around patient and caregiver goals* *Attention to providing comfort/support and managing behaviors to improve quality of life*	*“The doctor (…) she encouraged me to [do things with my granddaughters]*… *So I took that to heart and started doing some in‐person things and trying to do that more often (*…*).”* Patient B‐Site 3
Multi‐complexity	*Care Coordination* *Education around treatments/medications for one disease affecting symptoms of another* *Connecting patient well‐being with caregiver well‐being*	*“You know, (they said), if you wear your oxygen, you know, you are gonna remember more, and believe it or not he has said to me, “You know, hey, I think this might be working a little bit ‘cause I remember a little bit more’ and he has. I noticed a difference (…).”* Caregiver D‐Site 5

#### 3.5.2 Challenges associated with the visit

Despite many perceived benefits, some participants also noted challenges that negatively impacted perceptions of the quality and value of the GRECC Connect telemedicine visit. Three major challenges were: (1) difficulty accessing services recommended during the visit due to systems issues or miscommunication regarding who was to initiate contact regarding the service; (2) not receiving as much information as desired mostly around treatments or activities to slow the progression of their disease; and (3) uncertainty related to instructions and follow‐up plan, particularly related to whether they should be contacting their primary care or GRECC Connect clinician with questions. This last challenge is illustrated by the following quote from a caregiver expressing her confusion post‐visit:

*“So, we're not taking it [the new medication prescribed from the GRECC Connect visit], and [the patient's primary care provider] knows that. [The GRECC Connect clinician] does not know that ‘cause I don't know if I'm supposed to stay in touch with her. It really wasn't made clear to me, or maybe I just didn't understand who I'm supposed to really talk to about [the patient] and this diagnosis.”* Caregiver E‐Site 1.

## 4 DISCUSSION

Telemedicine is a potential solution to the barriers many rural, older adults face accessing in‐person specialty care.^6^, ^8^, ^9^ Because much literature exists that demonstrates the feasibility and acceptability of the virtual modality for older adults,^13^, ^19^, ^23^, ^24^, ^27^ we chose to focus this work on the content of geriatric telemedicine visits to explore whether high‐quality geriatric care could, in fact, be delivered virtually. Overall, patients and caregivers were highly satisfied with GRECC Connect telemedicine. They described several benefits including improved health and increased knowledge, confidence, empowerment, hope, and support. The content of visits and benefits perceived by patients and caregivers are well‐aligned with the Age‐Friendly Health Systems model^30^ and Geriatric 5Ms framework.^31^ The 5 M domains (the original 4Ms ‐ medication, mentation, mobility, matters most, plus the 5th “M” multi‐complexity) are viewed by experts in geriatric health care delivery as essential components of high‐quality care.^31^, ^33^ GRECC Connect visit content discussed by patients and caregivers aligns with the 5Ms, which suggests participants experienced quality health care visits. This is supported further by patients' and caregivers' association of these 5 M‐aligned aspects of the visit with many of the benefits they experienced, such as a discussion of a cognitive evaluation (a focus on Mentation) resulting in greater knowledge and confidence in the part of the caregiver.

At the same time, some experienced challenges that detracted from the experience—namely, not being able to access services recommended during telemedicine visits due to communications, systems, or geographic barriers (e.g., rurality), lack of clarity on medical advice following the visit and who to contact with questions, and a desire for more information on what they could do to slow or stop the progression of their disease (mostly dementia).

Lack of access to health care services is a system‐level issue and is prevalent across health care organizations,^1^, ^42^ not just the VA. Indeed, geriatric telemedicine exists to address some of these access issues. Insofar as telemedicine visit‐generated recommendations are considered key to supporting a patient and caregiver's health and wellbeing, it is important that efforts to provide care do not stop at the referral/recommendation stage. Some aspects of this are, of course, more readily addressed than others. For example, miscommunication around who should be initiating contact for a recommended follow‐up service could be mitigated with after‐visit summaries that address this. Other access issues are more challenging. For example, the interviews for this study were conducted during the COVID‐19 pandemic when many VA and community‐based services were severely restricted due to public health concerns and lack of staff. This contributed to some participants' inability to access needed follow‐up services.

After‐visit summaries could also address the lack of clarity and confusion some patients and caregivers experienced once their GRECC Connect telemedicine visit had concluded, particularly around who to contact with questions. While this may be a challenge with specialty care in general, it seems especially important to address for this older population^16^ as they are more likely to have numerous medical providers and health care appointments and hearing, visual, and cognitive impairments that may exacerbate the ability to absorb and recall information.^43^, ^44^

Patients' desire to have received more information to gain control of their health is also not likely limited to geriatric specialty care visits but may happen with other types of health care visits, especially when related to something as sensitive and potentially devastating as the loss of one's memory. It is not always possible to provide a diagnosis and some diseases have few evidence‐based treatments. This causes a potentially frustrating situation for patients, caregivers, and clinicians alike. Setting expectations and acknowledging the limits of what is known about specific medical conditions may be helpful, in addition to focusing on what *can* be done, as patients and caregivers in our study described that this made them feel more empowered and hopeful.

Despite these challenges, our findings demonstrate it is possible to deliver high‐quality geriatric care virtually to older adults. It is important, however, to recognize the uniqueness of the GRECC Connect program. This program, established six years before the onset of the COVID‐19 pandemic, has invested substantially in the geriatric telemedicine service and ongoing evaluation and quality improvement.^45^, ^46^, ^47^ This VA national network of experts functions as a learning collaborative, meeting regularly to share lessons learned and best practices. The experience they have gained over the years has not only gone into improving their own practice but is available to others via recorded educational workshops, conference presentations, and toolkits.^48^, ^49^ These resources may help those who are new to geriatric telemedicine provide similar, high‐quality health care to their patients.

The findings of our study are limited by the small sample of older patient participants who were all White and male, despite attempts to recruit a more diverse group of interviewees. This is, in part, because most rural Veterans served by the VA are White and male,^50^ but may also be attributable to racial and socioeconomic disparities in telehealth use.^51^, ^52^ By virtue of our study objectives, we included only patients and caregivers who had participated in a telemedicine visit. Study participants likely differ in many ways from older, rural adults and caregivers who are unable or unwilling to engage in telemedicine. Rural, older adults are less likely to have reliable Internet^52^ and more likely to have sensory and cognitive impairments that impede the use of telehealth.^19^ Additionally, this population is less likely to have the technological devices or literacy needed to successfully participate in virtual medical visits.^20^, ^21^, ^22^ Therefore, additional research is needed not only to address the challenges identified to continue to improve geriatric telemedicine but to also ensure high‐quality geriatric care reaches *all* those who could benefit from it.

## 5 CONCLUSION

As dissemination of the Age‐Friendly Health Systems model continues both within and outside the VA, it is important to consider how telemedicine visits can be used to expand access to high‐quality, Age‐Friendly geriatric care, particularly for rural patients who may have difficulty accessing larger, urban medical centers. Our work demonstrates that GRECC Connect, a national VA tele‐geriatrics initiative, addresses the core domains of the Age‐Friendly model as reflected by the benefits perceived by rural older patients and their caregivers.

## FUNDING INFORMATION

This work was supported by funding from the United States (U.S.) Department of Veterans Affairs, Office of Rural Health, Veteran Rural Health Resource Center‐Salt Lake City for portfolio project FY21‐16035. Dr. Pimentel is supported by the Career Development Award IK2 HS00318 from the U.S. Department of Veterans Affairs Health Services Research and Development Service. The funders had no role in the preparation, review, or approval of the manuscript or decision to submit it for publication. The content of this manuscript is solely the responsibility of the authors and does not necessarily represent the official views of the U.S. Department of Veterans Affairs or the United States Government.

## CONFLICT OF INTEREST

The authors declare that there is no conflict of interest.
